# Priming the Kidney for Injury: Immune Consequences of Chronic Psychological Stress

**DOI:** 10.34067/KID.0000001187

**Published:** 2026-06-25

**Authors:** Gillian C. Kelly, Cailin E. Kellum, Jennifer S. Pollock

**Affiliations:** CardioRenal Physiology and Medicine, Division of Nephrology, Department of Medicine, University of Alabama at Birmingham, Birmingham, Alabama

**Keywords:** immunology, kidney

Stress is a critical response state provoked by physical, psychological, and environmental challenges. Between 2019 and 2022, stress became more prominent in the United States.^1^ The shift from an acute, adaptive response to a chronic state promotes sustained biological consequences.^2^ While acute stress responses are essential for adaptability and survival, chronic psychological stress (CS) is characterized by prolonged activation of stress-response pathways and detrimental to longer-term health.^2^ Chronic psychological stress is recognized as a contributing risk factor in an estimated 75%–90% of disease states, underscoring its broad effect on human health.^2^ There is a growing recognition that CS shapes immune-mediated outcomes across organ systems.^3^ The immune system is also now well established as a central contributor to both AKI and CKD.^4^ Immune cells regulate renal injury, repair, and fibrotic remodeling, exerting effects that may be protective or pathogenic depending on context, timing, and cellular phenotype.^4^ Stress-induced immune alterations have been documented in the brain, liver, and spleen, and gut, yet the kidney has largely been absent from these studies.^5^ The study by Rastegar *et al.* directly tackles this gap by examining renal immune responses in a well-established mouse model of CS.^6^

CS has long been associated with immune dysregulation and adverse health outcomes. Seminal studies beginning in the 1980s demonstrated that stress alters leukocyte distribution, antiviral immunity, and enhances inflammatory signaling.^7^ Subsequent work established that stress exerts lasting effects on both innate and adaptive immune responses.^3^ The bidirectional communication between the brain-kidney axis through autonomic, endocrine, and immune pathways has received more focus in recent years.^8^ Using a mouse model of CS, Rastegar *et al.* demonstrate that CS adversely affects health and drives kidney-specific immune remodeling in the absence of overt renal disease or traditional risk factors.^6^ CS exposure resulted in greater weight gain, reduced fur quality, renal inflammation, and marked shifts in renal immune composition, including reduced CD4^+^ *T* cell abundance with enrichment of both *T* helper 17 (Th17) and regulatory *T* cell (Treg) subsets, as well as increased macrophages within kidney tissue. These immune changes were accompanied by elevated intrarenal TNF-*α* and increased circulating biomarkers of kidney injury, including cystatin C and neutrophil gelatinase-associated lipocalin, providing functional relevance to the immune cellular findings. The coexistence of Th17 expansion, typically associated with renal inflammation and fibrosis, with increased Tregs suggests a dynamic immune imbalance in which competing inflammatory and regulatory signals are engaged within the kidney exposed to stress. Together, these findings support a link between CS and early, kidney-specific immune and inflammatory changes that may prime the kidney for risk of injury and dysfunction (Figure 1).

**Figure 1:**
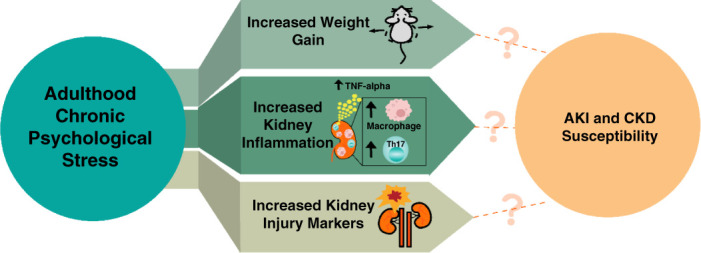
**Proposed mechanisms of how CS in adulthood may lead to AKI and CKD susceptibility.** CS exposure in mice resulted in increased weight gain, which is a risk factor for CKD. In addition, CS leads to increased kidney inflammation marked by Th17 expansion, macrophage accumulation, and elevated TNF-*α*. Finally, CS increased circulating kidney injury markers, cystatin C, and NGAL. Together, these pathways reveal kidney-specific immune, inflammatory, and physiological changes that may prime the kidney for acute and chronic disease severity. CS, chronic psychological stress; NGAL, neutrophil gelatinase-associated lipocalin; Th17, *T* helper 17.

An important strength of this work is the use of a psychosocial CS paradigm. Stress is a heterogeneous exposure, encompassing physical stressors, neglect, and social adversity. Social stressors are particularly relevant to human disease because they are strongly associated with depression-related and anxiety-related behaviors and robust engagement of immune and inflammatory pathways. Models of CS may therefore more closely reflect clinically relevant stress exposures than purely physical stress protocols.

Despite the important insights provided by this study, several key questions remain that will be critical to address in future work. Notably, the chronic social defeat stress paradigm used here is largely restricted to male mice, reflecting the sex-specific nature of the model itself. Given well-documented sex differences in stress responsivity, immune regulation, and renal disease progression, extending these findings to females will require the use of alternative, etiological relevant stress paradigm. For example, using the overcrowding stress protocol may be more relevant with female patients.^9^ Although shifts in renal immune cell proportions were clearly demonstrated, the origins and functional roles of these populations remain undefined. Distinguishing between resident and infiltrating immune cells and determining whether stress-induced immune responses serve protective, compensatory, or pathogenic roles within the kidney will be essential for understanding their relevance to injury risk. Finally, the observed increase in renal macrophages raises important questions regarding macrophage heterogeneity because distinct subsets can exert divergent effects on inflammation, tissue repair, and fibrosis. Further characterization of these populations will be necessary to clarify how CS shapes renal immune landscapes and influences long-term kidney health.

In perspective of broader clinical context, stress-induced immune remodeling within the kidney may have important implications for susceptibility to both AKI and CKD. AKI and CKD are increasingly recognized as immunologically driven conditions, in which the pre-existing immune status can influence the severity of injury, the capacity for repair, and progression toward fibrosis.^10^ The proinflammatory and plastic immune environment observed after CS, characterized by Th17 expansion, macrophage accumulation, and elevated TNF-*α* alongside increased Treg representation, may prime the kidney for maladaptive responses to subsequent insults, such as ischemia, infection, or nephrotoxic exposure. In this framework, CS establishes a vulnerable immunologic landscape that lowers the threshold for injury and impairs resolution. Over time, repeated or unresolved immune activation may promote the transition from acute injury to chronic dysfunction. Integrating CS into experimental models of AKI and CKD may therefore be critical for understanding disease heterogeneity and identifying stress-responsive immune pathways that modify renal resilience and longer-term outcomes.

## References

[1] Terlizzi EP Zablotsky B. National Health Statistics Reports, Number 213, November 4, 2024. National Health Statistics Reports Number, Vol 213; 2019. Accessed December 30, 2025. https://www.cdc.gov/nchs/products/index.htm.

[2] Agorastos A Chrousos GP. The neuroendocrinology of stress: the stress-related continuum of chronic disease development. Mol Psychiatry. 2021;27(1):502–513. doi:10.1038/s41380-021-01224-9 34290370

[3] Rohleder N. Stress and inflammation – the need to address the gap in the transition between acute and chronic stress effects. Psychoneuroendocrinology. 2019;105:164–171. doi:10.1016/J.PSYNEUEN.2019.02.021 30826163

[4] Motrenikova M Boyanov K Bojinova N Bivolarska A. Stress pathways in chronic kidney disease: linking cortisol, oxidative stress, and inflammation. Antioxidants (Basel). 2025;14(10):1259. doi:10.3390/ANTIOX14101259 41154568 PMC12561467

[5] Chan KL Poller WC Swirski FK Russo SJ. Central regulation of stress-evoked peripheral immune responses. Nat Rev Neurosci. 2023;24(10):591–604. doi:10.1038/s41583-023-00729-2 37626176 PMC10848316

[6] Rastegar TF Patel SK Kapoor R, . Chronic psychologic stress in mice induces kidney inflammation. Kidney360. 2026;7(6):1201–1211. doi:10.34067/KID.0000001111 41538290 PMC13337208

[7] Studies on the Effect of Social Stress on Measures of Disease Resistance in Laboratory Mice, 1982. Accessed December 30, 2025. https://psycnet.apa.org/record/1983-05146-001

[8] Xie Z Tong S Chu X Feng T Geng M. Chronic kidney disease and cognitive impairment: the kidney-brain axis. Kidney Dis. 2022;8(4):275–285. doi:10.1159/000524475 PMC9386403 36157262

[9] Furman O Tsoory M Chen A. Differential chronic social stress models in male and female mice. Eur J Neurosci. 2022;55(9-10):2777–2793. doi:10.1111/ejn.15481 34587653

[10] Sato Y Yanagita M. Immune cells and inflammation in AKI to CKD progression. Am J Physiol Renal Physiol. 2018;315(6):F1501–F1512. doi:10.1152/ajprenal.00195.2018 30156114

